# Feasibility, Safety and Efficacy of Enhanced Recovery after Living Donor Nephrectomy: Systematic Review and Meta-Analysis of Randomized Controlled Trials

**DOI:** 10.3390/jcm10010021

**Published:** 2020-12-23

**Authors:** Apostolos Prionas, Charles Craddock, Vassilios Papalois

**Affiliations:** 1 Department of Surgery and Cancer, Imperial College London, London SW7 2AZ, UK; vassilios.papalois@nhs.net; 2 Department of General Surgery, Barking, Havering and Redbridge University Hospitals NHS Trust, London RM7 0AG, UK; charlescraddock@gmail.com

**Keywords:** living donor nephrectomy, enhanced recovery after surgery, living organ donation, postoperative morbidity, length of hospital stay, patient experience, patient quality of life

## Abstract

This meta-analysis aims to compare enhanced recovery after surgery (ERAS) vs. standard perioperative practice in the management of living kidney donors. Primary endpoints included mortality, complications, length of stay (LOS) and quality of life after living donor nephrectomy. Medline, Embase, Scopus, Cochrane and Web of Science databases were searched. In total, 3029 records were identified. We then screened 114 full texts. Finally, 11 studies were included in the systematic review corresponding to 813 living donors. Of these, four randomized controlled trials were included in the meta-analysis. ERAS resulted in shorter LOS (95CI: −1.144, −0.078, I^2^ = 87.622%) and lower incidence of post-operative complications (95CI: 0.158, 0.582, I^2^ = 0%). This referred to Clavien–Dindo I-II complications (95CI: 0.158, 0.582, I^2^ = 0%). There was no difference in Clavien–Dindo III-V complications (95CI: 0.061,16.173, I^2^ = 0%). ERAS donors consumed decreased amounts of narcotics during their hospital stay (95CI: −27.694, −8.605, I^2^ = 0%). They had less bodily pain (95CI: 6.735, 17.07, I^2^ = 0%) and improved emotional status (95CI: 6.593,13.319, I^2^ = 75.682%) one month postoperatively. ERAS protocols incorporating multimodal pain control interventions resulted in a mean reduction of 1 day in donors’ LOS (95CI: −1.374, −0.763, I2 = 0%). Our results suggest that ERAS protocols result in reduced perioperative morbidity, shorter length of hospital stay and improved quality of life after living donor nephrectomy.

## 1. Introduction

Living donor renal transplantation (LDRT) is the optimal form of renal replacement therapy (RRT) for suitable patients with end stage renal disease (ESRD). When compared to the alternative of dialysis therapy, either as hemodialysis or peritoneal dialysis, it is associated with substantial improvement in quality of life (QoL), reduced mortality and morbidity and increased cost-effectiveness [1]. In comparison to deceased donor renal transplant (DDRT), transplantation from living donors results in improved outcomes, including prolonged graft and recipient survival [2,3].

Living kidney donation is not associated with significant long-term health risks. O’Keeffe et al., in their 2018 meta-analysis published in *Annals of Internal Medicine*, found that, apart from a low absolute risk for future development of ESRD (1 case per 1000 person-years) and pregnancy complications, living kidney donors have no increased risk for other major chronic diseases, long-term morbidity and mortality [4].

Despite the low risks for donors, the benefits to recipients and to society at large, the rates of LDRT remain low compared to dialysis therapy and DDRT, internationally. In 2019 across Europe, only 9.94 LDRTs per million population (pmp) were performed [5]. To put this in context, the equivalents were 854 patients pmp in dialysis therapy and 26 DDRTs pmp [5]. Improving incentives for living donation should, therefore, be a priority in modern clinical medicine. 

Living organ donors are healthy individuals coming forward altruistically to help their loved ones. They derive no physical benefit from donating. It is, therefore, imperative that the safety and the efficacy of the donation process meet the highest standards. The concept of enhanced recovery after surgery (ERAS) has revolutionized surgical practice in recent years since its introduction by Henrik Kehlet in the 1990s. ERAS is about improving outcomes and reducing the duration of postoperative recovery. It aims to ensure that patients receive evidence-based care at the right time. Compared to traditional perioperative management, ERAS represents a fundamental shift in the process of care, by incorporating multiple interventions that attenuate surgical stress, maintain physiological function and expedite return to baseline. While each intervention has a small effect, all together they have a stronger synergistic impact. Although the ERAS principles were originally developed and integrated for colorectal surgery, they have been adopted by multiple surgical specialties [6].

The present study aims to systematically review the existing evidence on the feasibility, the safety and the efficacy of ERAS compared to traditional perioperative care in the management of living kidney donors. In addition, we aim to summarize the different preoperative, intraoperative and postoperative ERAS interventions and streamline them in order to fulfil the need for one state-of-the-art ERAS protocol for living donor nephrectomy (LDN).

## 2. Materials and Methods

### 2.1. Protocol and Registration

The protocol for this meta-analysis was designed, reviewed, standardised and registered in the Imperial College London Registry (Department of Surgery and Cancer) prior to the initiation of the study. This study is part of a thesis project for the acquisition of the degree of Master’s in Science (MSc) in Surgical Innovation. The protocol can be provided upon communication with the authors’ team.

### 2.2. Eligibility Criteria

The present systematic review aims to address a specific research question: whether the implementation of sequential ERAS interventions is safe and efficient in the management of living kidney donors according to the existing evidence. The PICO breakdown of this research question is presented below:

- Patients: living kidney donors.
- Interventions: sequential ERAS interventions or protocols.
- Comparison: standard care.
- Outcomes: postoperative morbidity (complication rates), postoperative mortality, length of stay (LOS), unplanned readmissions and QoL after surgery.

Therefore, the inclusion criteria for the present systematic review were defined as:

1. Randomized Controlled Trials (RCTs), retrospective, prospective, interventional and comparative studies.
2. Studies that enrolled adult patients undergoing living donor nephrectomy.
3. Studies involving sequential ERAS interventions (including ERAS protocols). ERAS intervention was defined as any intervention that aims to attenuate surgical stress, maintain physiological function and expedite return to baseline. For a study to be deemed eligible for inclusion, at least two sequential ERAS interventions were required (i.e., one preoperative and one intraoperative intervention or one intraoperative and one postoperative intervention etc.).
4. Studies in which standard care was the main comparator.
5. Studies that reported complication rates, mortality, length of stay, unplanned readmissions and QoL data in the ERAS and the control groups.

Specific exclusion criteria were also applied in order to approach the research question, defined as:

1. Animal, cadaveric studies, commentaries, editorials, case repots, reviews, meta-analyses, protocols, conference abstracts for which a detailed study report (published or unpublished) was not available and studies that did not include sequential ERAS interventions.
2. Studies that enrolled pediatric patients or patients undergoing nephrectomy for other reasons (i.e., cancer) and studies that did not enroll or excluded participants with major complications.
3. Studies involving a single intervention. Since the present study aims to determine the cumulative effect of sequential ERAS interventions (care bundles) in the management of renal transplant patients, studies that involved solitary interventions were excluded.
4. Studies with no comparator and studies that did not report the abovementioned outcomes.

### 2.3. Information Sources

After establishing a detailed and thorough search strategy, Medline, Embase, Scopus, Cochrane Library and Web of Science databases were searched from the time of their design to the 10th of May 2020. Grey literature was included to mitigate the risk of publication bias. Only detailed reports of unpublished studies were considered eligible for inclusion.

### 2.4. Search

The full electronic searches for the Medine and Embase Databases (two of the databases searched) are shown in the Supplementary Tables S1 and S2, respectively.

### 2.5. Study Selection

All references were imported into reference management software (Endnote Library- Endonote X9, Clarative Analytics, Philadelphia, United States). Duplicates were removed. Two independent reviewers screened the titles and abstracts and decided which studies would proceed in the full-text eligibility screen. There were three conflicts at this stage; two were resolved with discussion and one by the vote of the third author. Full texts were screened for eligibility independently by two reviewers. Following selection for inclusion, the studies’ qualities were assessed (study size, length of follow up and risk of bias). Due to the observed high risk of bias within and across the non-randomized studies, only RCTs were included in the meta-analysis part of the systematic review.

### 2.6. Data Collection Process

Data were extracted from every study individually using a predefined standard operation spreadsheet. This was piloted and tailored appropriately. There was no communication with authors for the purpose of obtaining or confirming data.

### 2.7. Data Items

Comparative Data between ERAS and standard care groups were extracted for the following domains:

1. Living Donors Characteristics: age in years (mean ± SD / median, (range)), gender (proportion of male donors, % percentage), Body Mass Index (BMI) in kg/m^2^, left nephrectomy (proportion in the study population, % percentage), smoking history (proportion in the study population, % percentage) and comorbidities (proportion of reported comorbidities in the study population, % percentage).
2. ERAS and standard care interventions: preoperative, intraoperative and postoperative ERAS and standard care interventions (qualitative data).
3. Donors Outcomes:
  - Surgical mortality (proportion in the study population, % percentage);
  - Postoperative complications including severity (proportion of Clavien–Dindo I- II and Clavien–Dindo III-V complications in the study population, % percentage);
  - Hematocrit drop postoperatively (mean ± SD);
  - need for postoperative blood transfusion (proportion in the study population, % percentage);
  - LOS (mean ± SD / median, (range));
  - Unplanned readmissions (proportion in the study population, % percentage),
  - Reasons for readmissions (qualitative data);
  - Adverse events (qualitative data);
  - QoL after surgery (pain scores in postoperative days 1 and 2 (mean ± SD), morphine requirements in mg during the first 24 h, from 24 to 48 h postoperatively and cumulatively during the first 48 h or the entire hospitalization (mean ±SD). To further assess living donors’ QoL after surgery data on donors scores’ in all the different dimensions of the Short Form-36 (SF-36) questionnaire preoperatively and one month postoperatively, were extracted (mean ±SD) [7]).

Furthermore, data on the characteristics and the outcomes of the recipients were extracted in order to capture any adverse effects of the donors’ ERAS interventions on the recipients. Such adverse effects were not detected and, therefore, these data are not reported. 

### 2.8. Risk of Bias in Individual Studies

The risk of bias in individual studies was assessed using research tools, appropriate for the different study types, as recommended by the Cochrane Collaboration. These were the “Risk Of Bias In Non-randomized Studies of Interventions” (ROBINS-I) assessment tool and the “Revised Cochrane Risk-of-Bias tool for randomized trials” (RoB 2) [8,9]. The assessment of risk of bias was used to determine which studies would proceed to the meta-analysis stage of the systematic review. The risk of bias assessment was done on two levels (study and outcome level assessment).

### 2.9. Summary Measures

In dichotomous variables, Odds Ratio (OR) was used to summarize the difference between the groups. For comparison of continuous variables, the difference in means was used.

### 2.10. Synthesis of the Results

The meta-analyses results were reported using 95% CI and displayed graphically using forest plots. The results of the studies were combined in meta-analyses using standard random effects models, from which estimates of the average mean difference (ERAS-standard care) or OR (ERAS vs. standard care) were obtained, with 95% CI. To assess heterogeneity, I2 was obtained. All analyses were conducted in the OpenMetaAnalyst software with a significance level of 0.05. 

### 2.11. Risk of Bias Across Studies

We aimed to minimize risk of bias across studies. On a protocol level, the search included both published and unpublished literature with no language and time restrictions to minimize publication bias. The different risks of bias domains were assessed individually for every study and collectively across the different study types (nonrandomized studies Vs RCTs). The risk of bias in the majority of the relevant domains was high across the nonrandomized studies. Only RCTs were included in the meta-analysis. This was also done in an effort to mitigate the risk of selective reporting bias across studies.

### 2.12. Additional Analyses

Heterogeneity was anticipated. The PICO question of the study was heterogenous. ERAS and standard care interventions were anticipated to vary across the included studies. The statistical heterogeneity was explored when feasible.

## 3. Results

### 3.1. Study Selection

**Figure 1:**
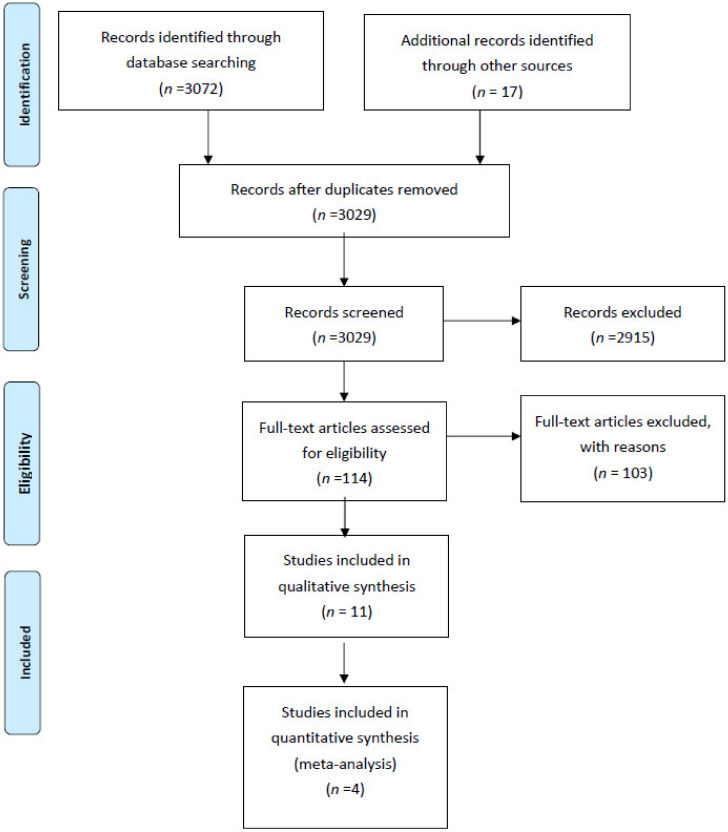
Systematic review flow diagram.

Figure 1 illustrates the study’s flow diagram. In total, 3072 records were identified through database screening and 17 through other sources. After removal of duplicates, 3029 abstracts were screened for inclusion in the systematic review. Of these, 2915 were excluded. In total, 114 full text articles were assessed for inclusion. Of these, 103 articles were excluded (10 studies involving ERAS protocols for renal transplant recipients; 27 conference abstracts with either insufficient information or duplicated data; 8 commentaries, systematic reviews, study protocols; 3 because patients with major complications were excluded; 3 because the relevant outcome data were not reported; 1 because there was no comparator; 3 because paediatric patients were enrolled; 3 because patients undergoing nephrectomy for cancer were included; 45 because they involved solitary interventions). Overall, 11 studies, 4 RCTs and 7 non-randomized cohort studies were included in the present systematic review [10,11,12,13,14,15,16,17,18,19,20].

### 3.2. Study Characteristics and Risk of Bias

Table 1 and Table 2 illustrate the characteristics of the studies included in the present systematic review. These correspond to a population of 813 living kidney donors. The summative risk of bias assessment is also presented. The detailed risk of bias assessments for the individual studies can be found in the Supplementary Files 1–11.

### 3.3. Results of Individual Studies

Table 3 illustrates the baseline characteristics of the living kidney donors included in the present systematic review. None of the studies reported donor comorbidities. Table 4 summarizes the ERAS interventions presented across the different studies. The donors’ outcomes are reported in Table 5, Table 6, Table 7, Table 8 and Table 9. None of the studies reported adverse events. Studies were excluded from the corresponding tables when they did not report any relevant data.

### 3.4. Synthesis of Results

#### 3.4.1. Qualitative Synthesis

Eleven studies, four RCTs and seven cohort/case-control studies, were included in the present systematic review. These corresponded to a population of 813 living donors. Donors in the ERAS and the standard care cohorts were in their majority young and healthy; no significant comorbidities were reported. The ERAS protocols presented in the included studies incorporated a variety of interventions. With regard to preoperative interventions, outpatient consultation for setting recovery goals [10,12,13,17,19,20], carbohydrate loading (with avoidance of peri-operative fasting) [10,12,13,15,19,20] and pre-emptive analgesia loading [11,19,20] were the most common ERAS elements encountered. Intraoperatively, the infiltration of local anaesthetic in the epidural space [12,13,15,16], the use of Transversus Abdominis Plane (TAP) blocks [19,20], and the insertion of local anaesthetic wound infiltration catheters [10,18] were the three most prominent ERAS interventions. Postoperatively, initiation of nutrition [10,12,13,17,19,20] and mobilization [10,12,13,17,20] from POD0 were common ERAS elements. With regard to postoperative pain management, nonsteroid anti-inflammatory drugs (NSAIDs) were the basic ERAS analgesic regime [11,13,14,16,19,20]. The administration of local anaesthetic through an epidural catheter [12,13,15] or through a wound infiltration catheter [10,15,18] was used to complement analgesic medications in the included studies. Three out of the eleven studies included in our systematic review defined specific clinical discharge criteria in their ERAS protocols [12,13,20]. With regard to the safety of the ERAS protocols, none of the included studies reported any perioperative deaths. ERAS donors tended to have lower overall incidence of postoperative complications compared to standard care donors’ [13,15,17]. This primarily referred to Clavien–Dindo I-II complications; Clavien–Dindo III-V were not reported [13,15,17]. Adverse events were also not reported. The use of NSAIDs did not result in haemorrhagic or nephrotoxic complications. Significant perioperative drops in ERAS donors’ haematocrit or rises in creatinine were not encountered [11,14]. ERAS resulted in improved quality of life after surgery (postoperative pain, narcotics consumption, and SF-36 questionnaire).

#### 3.4.2. Quantitative Synthesis

Four randomized controlled trials, corresponding to a population of 343 living kidney donors, were included in the present meta-analysis [11,13,14,15]. With regard to donors’ characteristics, between the ERAS and standard care groups, there were no significant differences for age (95CI: −3.528, 1.061) (*p* = 0.292) (I^2^ = 0%, Het. *p* = 0.666), male gender (95CI: 0.347, 1.125) (*p* = 0.117) (I^2^ = 22.502%, Het *p* = 0.276), BMI (95CI: −1.358, 1.040) (*p* = 0.795) (I^2^ = 34.364%, Het *p* = 0.206) and left sided nephrectomies (95CI: 0.261, 1.347) (*p* = 0.212) (I^2^ = 0%, Het *p* = 0.640). Only one study assessed the donors’ smoking histories and found no significant difference between the ERAS and the standard care groups [15].

##### Safety

**Figure 2:**
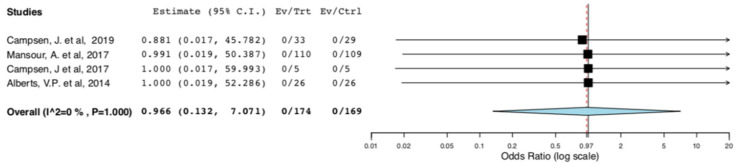
Surgical Mortality: Random Effects Meta-Analysis. (ERAS vs. Standard Care).

No deaths were reported in the included studies. Therefore, there was no difference in donors’ surgical mortality between the ERAS and the standard care groups (95CI: 0.132, 7.071) (*p* = 0.973) (I^2^ = 0%, Het *p* = 1) (Figure 2).

**Figure 3:**
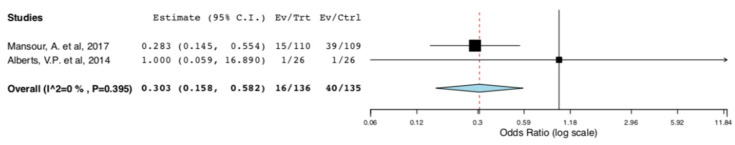
Surgical Morbidity (Overall Postoperative Complications): Random Effects Meta-Analysis. (ERAS vs. Standard Care).

**Figure 4:**
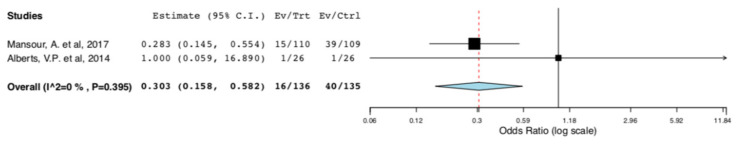
Surgical Morbidity (Clavien–Dindo I-II complications): Random Effects Meta-Analysis. (ERAS vs. Standard Care).

**Figure 5:**
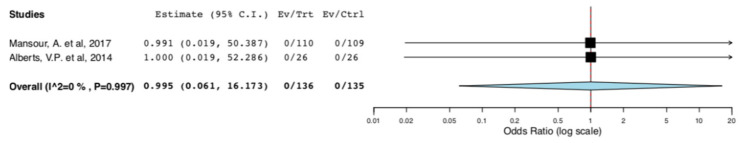
Surgical Morbidity (Clavien-Dindo III-V complications): Random Effects Meta-Analysis. (ERAS vs. Standard Care).

With regard to perioperative morbidity, two studies reported the incidence and the severity of postoperative complications. Figure 3 illustrates that ERAS donors had lower incidence of overall complications when compared to donors receiving standard care (95CI: 0.158, 0.582) (*p* < 0.001) (I^2^ = 0%, Het *p* = 0.395). The lower incidence of complications in the ERAS group accounted for Clavien–Dindo I-II complications (95CI: 0.158, 0.582) (*p* < 0.001) (I^2^ = 0%, Het *p* = 0.395), as illustrated in Figure 4. There was no difference in Clavien–Dindo III-V postoperative complications between the donors receiving enhanced recovery and those receiving traditional care (95CI: 0.061,16.173) (*p* = 0.997) (I^2^ = 0%, Het *p* = 0.997) (Figure 5).

##### Efficacy

**Figure 6:**
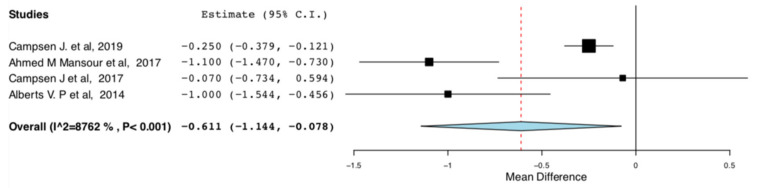
Length of Hospital Stay: Random Effects Meta-Analysis. (ERAS-Standard Care).

Length of Stay: As shown in Figure 6, donors in the ERAS groups had shorter length of hospital stay compared to the standard care groups (95CI: −1.144, −0.078) (*p* = 0.025) (I^2^ = 87.622%, Het *p* < 0.001). There is high heterogeneity in this meta-analysis (I^2^ = 87.622%).

Only one RCT reported unplanned donor readmissions after hospital discharge [11]. There was no difference in readmission rates between donors receiving ERAS and the standard care groups in this study [11].

QoL after Surgery: With regard to postoperative pain, two RCTs assessed the donors’ pain scores on POD1 and POD2 [13,15]. There were no significant differences between the ERAS and standard care groups on POD1 (95CI: −2.083, 0.229) (*p* = 0.116) (I^2^= 85.97%) and POD2 (95CI: −5.388, 3.294) (*p* = 0.636) (I^2^ = 98.136%, Het. *p* < 0.001).

**Figure 7:**
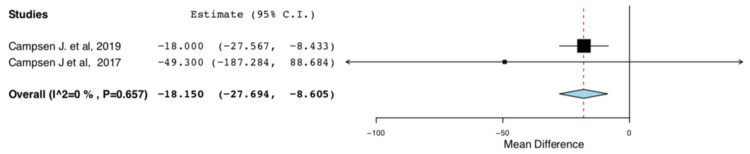
Cumulative Morphine Consumption during Hospital Stay: Random Effects Meta-Analysis. (ERAS-Standard Care).

Cumulative morphine consumption for the first 48 h post op or throughout the entire hospitalization was assessed by two RCTs. ERAS donors consumed less morphine in comparison to donors receiving traditional perioperative care (95CI: −27.694, −8.605) (*p* < 0.01) (I^2^ = 0%, Het *p* = 0.657) (Figure 7).

Two RCTs reported donors’ responses in the Short Form-36 (SF-36) questionnaire at two different time points, preoperatively and postoperatively. Between the ERAS and the standard care groups, there were no differences in donors’ scores in any of the domains of the questionnaire during the preoperative period. Postoperatively, there were no differences between the two groups in the domains of physical function (95CI:−1.047, 3.294) (*p* = 0.636) (I^2^ = 98.136%, Het *p* < 0.001), role physical (95CI:−9.706, 18.697) (*p* = 0.535) (I^2^ = 92.544%, Het *p* < 0.001), general health (95CI:−12.320, 13.742) (*p* = 0.915) (I^2^ = 95.785%, Het *p* < 0.001), vitality (95CI:−8.755, 9.960) (*p* = 0.9) (I^2^ = 82.567%, *p* = 0.017), social functioning (95CI:−0.034, 7.072) (*p* = 0.052) (I^2^ = 56.998%, Het *p*= 0.127) and mental health (95CI:−5.399,2.258) (*p* = 0.421), (I^2^ = 76.123%, Het *p* = 0.041).

**Figure 8:**
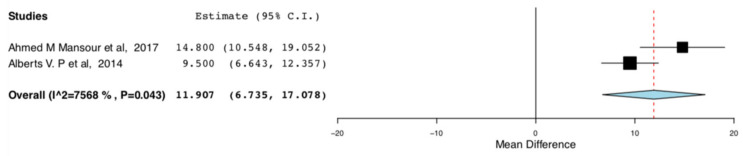
Postoperative SF-36 scores (Bodily pain): Random Effects Meta-Analysis (ERAS-Standard Care).

**Figure 9:**
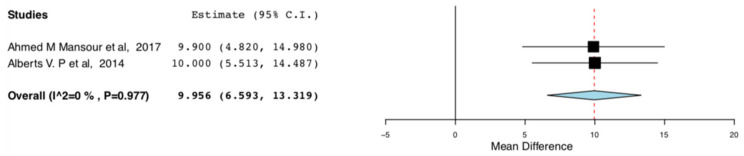
Post-operative SF-36 scores (Role emotional): Random Effects Meta-Analysis. (ERAS-Standard Care).

The enhanced recovery donors scored higher in the domains of bodily pain (95CI:6.735, 17.07) (*p* < 0.001) (I^2^ = 0, Het *p* = 0.515) and role emotional (95CI: 6.593,13.319) (*p* < 0.001) (I^2^ = 75.682, Het *p* = 0.043), compared to donors receiving standard care. This means that the donors receiving ERAS had less bodily pain and improved emotional status, one month postoperatively. The relevant results are shown in Figure 8 and Figure 9**,** respectively.

##### Additional Analyses

**Figure 10:**
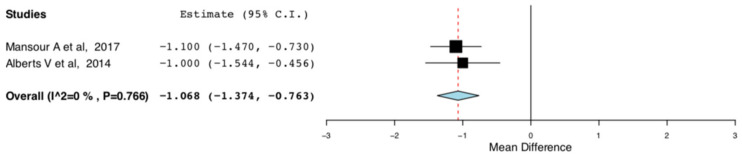
Length of Hospital Stay: Random Effects Meta-Analysis. (multimodal pain control ERAS interventions-Standard Care).

The random effect meta-analysis for the hospital length of stay between the ERAS and the standard care donors presented high heterogeneity. There was significant variation in the mean difference of the hospital lengths of stay between the ERAS and the standard care groups in the RCTs included in our meta-analysis. This was anticipated at a protocol level and was attributed to variations in the efficiency of the different ERAS protocols. More specifically, in the studies by Campsen J., et al., only IV/oral analgesia was used for perioperative pain control [11,14]. On the contrary, in the studies by Mansour A., et al. and Alberts V., et al., thoracic epidural was also used on top, for perioperative pain control [13,15]. This was done either by a one -off injection of long-lasting anaesthetic agent or by a continuous infusion of short-acting agent via an epidural catheter. In order to explore the abovementioned heterogeneity, we excluded the two studies that did not incorporate any alternative methods of perioperative pain control apart from analgesic medications. As it is illustrated in Figure 10, compared to traditional perioperative care, ERAS protocols incorporating multimodal pain control interventions result in shorter length of hospital stay by one day for the living kidney donors (95CI: −1.374, −0.763) (*p* < 0.01) (I^2^ = 0, Het *p* = 0.766).

#### 3.4.3. Internal Validity

Due to the observed high risk of bias, the non-randomized cohort studies were not included in the meta-analysis part of the systematic review. In order to assess our systematic review’s internal validity, we tested whether the results of the non-randomized studies were consistent with our meta-analyses findings. We pooled the results of the non-randomized cohorts along with the RCTs in subgroup meta-analyses (Supplementary File 12). We found that the results of the non-randomized studies were in agreement with the results of our meta-analysis. This further strengthened our study’s internal validity. We did not draw any additional conclusions from this analysis. 

## 4. Discussion

### 4.1. Summary of Evidence

To the authors knowledge, the present systematic review and meta-analysis of randomized controlled trials is the first to compare the safety and the efficacy of enhanced recovery pathways and traditional perioperative care in the management of living kidney donors. Our systematic review demonstrated the feasibility of enhanced recovery after living donor nephrectomy. Our meta-analysis showed that, compared to standard care, ERAS protocols result in reduced perioperative morbidity, shorter length of hospital stay and improved quality of life after living donor nephrectomy. Furthermore, by summarizing the available evidence corresponding to a population of 813 living donors, our study streamlined the collective published experience to a single state-of-the-art ERAS protocol for living donor nephrectomy.

The primary objective of this study was to determine the safety and efficiency of the implementation of ERAS in the management of living kidney donors. The safety of the donor is of paramount importance in donation surgeries. As anticipated, there was no difference in surgical mortality rates between the ERAS and the standard care groups in our study. None of the studies included in our systematic review reported perioperative deaths. More importantly, we found that ERAS donors had lower incidence of post-operative complications compared to donors receiving standard care. This primarily refers to minor complications (Clavien–Dindo I–II). There was no difference in the incidence of major complications (Clavien–Dindo III–V). These findings are in agreement with the existing evidence from the implementation of ERAS programs in colorectal surgery [21].

Our study also aimed to assess the efficiency of ERAS protocols following living donor nephrectomy. The living kidney donors receiving enhanced recovery had shorter length of stay compared to the traditional perioperative care groups. This is in agreement with data from the implementation of ERAS protocols in colorectal and noncolorectal abdominal surgical procedures [22,23]. The numerical reduction in LOS varied between the studies included in our meta-analysis. This reflects the variable levels of efficiency of the different ERAS protocols. Protocols incorporating multimodal pain control ERAS interventions resulted in greater LOS reduction. With regard to unplanned readmissions, among the RCTs included in our meta-analysis, only Campsen et al. looked into donor readmissions. They found no difference between the ERAS and the standard care groups [11]. Among the non-randomized cohorts included in our qualitative synthesis, 5 out of 7 studies reported a reduction in unplanned readmissions of living kidney donors following the establishment of an ERAS pathway. Only Kuo P., et al. reported a higher absolute number of readmissions in their ERAS cohort. This was not statistically significant and referred to a single patient readmitted immediately after hospital discharge with nausea [17]. This stresses the important role of antiemetics and prokinetics in ERAS protocols for abdominal operations. In the ERAS protocol implemented in the study by Kuo P., et al., there was no preoperative loading with antiemetics. The protocol included regular administration of ondansetron intraoperatively and only pro re nata (PRN) administration postoperatively [17]. Nevertheless, definitive conclusions with regard to donors’ readmissions following ERAS cannot be reached from the present systematic review.

Regarding quality of life after surgery, our meta-analysis did not show a significant difference in pain scores reported by the donors’ on POD1 and POD2 between the ERAS and the standard care groups. Nevertheless, ERAS donors consumed decreased amounts of narcotics throughout their hospital stay compared to standard care donors. Given the fact that the ERAS and standard care donors in the studies included in the corresponding meta-analysis had the same level of access to narcotics (narcotic administration PRN), this finding is likely to represent advanced pain management within the enhanced recovery pathways. This is further strengthened by the pain scores reported by the donors’ included in the non-randomized studies. Most importantly, our study showed that, compared to donors receiving standard care, donors receiving enhanced recovery after LDN scored higher on the domains of bodily pain and role emotional of the SF-36 questionnaire, one month postoperatively. This suggests that ERAS pathways mitigate long-term physical stress deriving from the nephrectomy operation in a greater extent compared to traditional perioperative care. ERAS protocols also seem to be better achieved by the donors. These findings are in agreement with evidence deriving from the implementation of ERAS protocols in other surgical fields [21,22,23].

The secondary objective of this study was to fulfil the need for a state-of-the-art ERAS protocols for living donor nephrectomy. This was done through summarizing and combining the different ERAS interventions. The main features of this protocol are the involvement of the donors in their care (setting recovery goals preoperatively, early mobilization and initiation of nutrition post-operatively), the minimization of the surgery’s physiological impact (pre-operative carbohydrate loading, minimally invasive surgery) and the use of multimodal pain control interventions (NSAIDS, nerve blocks, epidurals, On-Q pumps) with simultaneous avoidance of narcotics. Off course, all these interventions do not come without potential risks. Nephrotoxicity or haemorrhagic complications from the administration of NSAIDs and spinal cord compression secondary to an epidural hematoma are some of the potential harms. Despite the fact that these adverse events were not reported in any of the included studies, clinicians are advised to keep an open mind and tailor the protocol accordingly, based on the individual donor cases.

### 4.2. Limitations

The present study had several limitations. There was increased risk of bias across the nonrandomized studies included in our systematic review. In order to mitigate this risk, the nonrandomized studies were not included in the meta-analysis part of the review. This resulted in all the outcomes been assessed through quantitative synthesis of the results of four RCTs. Only two studies were used for some outcome analyses. Among the RCTs included in our meta-analysis, one had overall high risk of bias in a study level. This referred to bias in the selection of the reported results. The direction of bias was in favour of ERAS interventions. In an outcome level, the risk of bias in the selection of the reported results was relevant for quality of life data after surgery. The selective reporting bias was partially mitigated by the fact that only randomized prospective data were taken into consideration. Furthermore, we used the nonrandomized data to test the internal validity of our meta-analysis findings. With regard to consistency measures, the present meta-analysis demonstrated a high degree of statistical heterogeneity in two of the primary endpoints: LOS (I^2^ = 87.622%) and bodily pain one month postoperatively (I^2^ = 75.68%). The source of heterogeneity in the former meta-analysis was further explored and the underlying clinical etiology was found. The source of heterogeneity in the latter meta-analysis could not be further explored statistically. We believe that it most likely originates from variations in the efficiency of the different experimental and comparative interventions, across studies. The statistical heterogeneity in the random effect meta-analyses for all the remaining primary endpoints was very low (I^2^ = 0%).

## 5. Conclusions

Compared to traditional practice, ERAS protocols result in reduced perioperative morbidity, shorter length of hospital stay and improved quality of life after Living Donor Nephrectomy.

## Figures and Tables

**Table 1:** Characteristics and risk of bias of non-randomized studies included in the systematic review.

Authors, Year	Study Size (ERAS/Standard Care)	Length of Follow Up	Risk of Bias								
			Confounding	Selection of Participants	Classification of Interventions	Deviations from Intended Interventions	Missing Data	Measure-ment of Outcomes	Selection of the Reported Result	Overall	Direction of Bias
Brown, T., et al., 2020 [10]	81 (57/24)	365 days	Serious	Low	Low	Low	Low	Moderate	Moderate	Serious	Unpredictable
Ricotta, C., et al., 2019 [12]	76 (21/55)	No info	Critical	Moderate	Serious	No info	Low	Moderate	Serious	Critical	Favours Standard Care
Rege A., et al., 2016 [19]	79 (39/40)	30 days	Moderate	Low	Low	Low	Low	Moderate	Low	Moderate	Favours Standard Care
Waits S., et al., 2015 [20]	120 (60/60)	14 days	Serious	Low	Low	Low	Low	Low	Low	Serious	Unpredictable
Panaro F., et al., 2011 [18]	20 (10/10)	No info	Critical	Critical	Low	Low	Low	Low	Low	Critical	Unpredictable
Milan Z., et al., 2011 [16]	26 (12/14)	7 days	Low	Low	Moderate	Low	Low	Low	Low	Moderate	Towards null
Kuo P., et al., 2000 [17]	68 (41/27)	365 days	Critical	Serious	Low	Low	Low	Low	Low	Critical	Unpredictable

**Table 2:** Characteristics and risk of bias of randomized clinical trials included in the systematic review and meta-analysis.

Authors, Year	Study Size (ERAS/Standard Care)	Length of Follow Up	Risk of Bias							
			Randomization Process	Effect of Assignment to Intervention	Effect of Adhering to Intervention	Missing Outcome Data	Measurement of the Outcome	Selection of the Reported Result	Overall	Direction of Bias
Campsen, J., et al., 2019 [11]	62 (33/29)	30 days	Low	Low	Low	Low	Low	Some Concerns	Some Concerns	Favours ERAS
Ahmed M. Mansour, et al., 2017 [13]	219 (110/109)	365 days	Low	Some Concerns	Some Concerns	Low	Low	Low	Some Concerns	Unpredictable
Campsen, J., et al., 2017 [14]	10 (5/5)	30 days	Low	Low	Low	Low	Low	Low	Low	Towards null
Alberts V. P., et al. 2014 [15]	52 (26/26)	90 days	High	Some Concerns	Some Concerns	Low	Some Concerns	High	High	Favours ERAS

**Table 3:** Donors’ baseline characteristics.

Authors, Year	Age (in years)		Male Gender		BMI (in kg/m^2^)		Left Kidney Donation		Smoking History	
	ERAS	Standard Care	ERAS	Standard Care	ERAS	Standard Care	ERAS	Standard Care	ERAS	Standard Care
**Non-Randomized Studies**										
Brown, T., et al., 2020 [10]	46.2 ± 12.2	44.2 ± 12.2	27/57 (47.4%)	12/24 (50%)	26.6 ± 3.2	25.9 ±4.8	Not reported	Not reported	Not reported	Not reported
Ricotta, C., et al., 2019 [12]	55.5 ± 7.5	50.4 ± 9.1	4/21 (19.4%)	14/55 (25,45%)	Not reported	Not reported	Not reported	Not reported	Not reported	Not reported
Rege A., et al., 2016 [19]	47 (34–53.5)	45 (35.8–49.5)	27/39 (69.2%)	27/40 (67.5%)	25.9 (23.4–28.3)	26.4 (23.3–28.8)	Not reported	Not reported	Not reported	Not reported
Waits S., et al., 2015 [20]	41.3 (21–66)	39.2 (20–60)	20/60 (33.3%)	30/60 (50%)	27.4 (18–39)	25.9 (17–46)	Not reported	Not reported	Not reported	Not reported
Milan Z., et al., 2011 [16]	50.3 ±13.4	46.2 ±14.3	7/12 (58.3%)	9/14 (64.3%)	25.2 ± 2.6	25.4 ± 2.8	Not reported	Not reported	Not reported	Not reported
**Randomized Clinical Trials**										
Campsen, J., et al., 2019 [11]	43.8 ± 11	45.1 ±12.2	7/33 (21%)	12/29 (41%)	26.6 ±4.7	26.9 ± 3.2	8/33 (24%)	9/29 (31%)	Not reported	Not reported
Ahmed M. Mansour, et al., 2017 [13]	39.1 ± 10.12	41.1 ±11.2	36/110 (32.7%)	40/109 (36.6)	26.3 ±5.2	25.8 ±4.9	Not reported	Not reported	Not reported	Not reported
Campsen, J., et al., 2017 [14]	42.8 ± 6.5	37.8± 15.3	1/5 (20%)	4/5 (80%)	23.8 ± 2.1	28.7 ± 5.1	Not reported	Not reported	Not reported	Not reported
Alberts V. P., et al., 2014 [15]	55 ± 10	54 ± 11	9/26 (35%)	11/26 (42%)	26 ± 3	26 ±3	16/26 (62%)	20/26 (77%)	5/26 (19%)	3/26 (12%)

**Table 4:** Summary of ERAS Interventions.

Preoperative ERAS Interventions
1. Outpatient assessment of nutritional status, respiratory and physical performance. Dieticians and physiotherapists review, diet and exercise program if necessary.
2. Smoking cessation.
3. Outpatient consultation for expectation management, information on ERAS pathway, setting recovery goals and informed consent.
4. Carbohydrate drinks pre-op. Less than 2 h preoperative fasting. No maintenance intravenous fluids.
5. No preoperative sedatives.
6. Analgesia loading (paracetamol ± Gabapentin).
7. Prophylactic antibiotics.
8. Antiemetics loading (scopolamine patch).
9. Compression stockings for thromboprophylaxis.
**Intraoperative ERAS Interventions**
1. Thoracic epidural catheter placed to administer continuous epidural analgesia / local anaesthetic. Alternatively, single shot epidural. Long lasting local anaesthetic can be injected between T7-T10.
2. Urinary catheter placement.
3. No routine use of Nasogastric Tube.
4. Maintain normothermia—Upper Body air heating.
5. Transversus Abdominis Plane block before Trocars placement.
6. Laparoscopic Donor Nephrectomy/ Hand Assisted Laparoscopic Donor Nephrectomy.
7. Minimum Fentanyl/Hydromorphone use. IV Ketorolac, IV Paracetamol, IV Dexamethasone can be used for intraoperative analgesia.
8. Goal Directed Fluid Therapy with non-invasive cardiac output monitoring throughout the procedure (Crystalloids 1–3 mL/kg/h, fluid boluses to increase stroke volume, Mannitol/Furosemide to increase diuresis).
9. Heparin administration before the vascular clamp placement and protamine administration after the vascular clamp release.
10. Wound infiltration catheter placement for continuous administration of local anaesthetic. Alternatively, long lasting local anaesthetic injection in the subfascial plane.
11. Further antiemetic administration at the end of the operation (Ondansetron).
12. Removal of urinary catheter (and Nasogastric tube if used) before leaving theatre/in recovery.
**Postoperative ERAS Interventions**
1. Antibiotic Coverage for the Postoperative day 0 (POD0).
2. Thromboprophylaxis with compression stockings and enoxaparin.
3. Pre-emptive treatment of Nausea and Vomiting (Scopolamine patch, regular ondansetron, promethazine if needed).
4. No IV fluids. Start liquid diet on POD0 (carbohydrate drinks can be given). Build up diet as tolerated.
5. Start early mobilization on POD0. Gradually advance mobilization.
6. Postoperative analgesia through wound infiltration/epidural catheter initially and IV Paracetamol, IV Ketorolac ± Gabapentin. Minimum opioids (oxycodone/Tramadol) if necessary. Switch IV analgesics to oral when fluids are tolerated. Catheters (wound infiltration catheter/ epidural catheter) trial removal on POD1 (stop infusion for 6 h). Definitive removal of all catheters before POD2.
7. Aim for hospital discharge on POD2. Discharge Criteria: i) pain control with oral analgesics, ii) no complications requiring hospital care (normal temperature, stable hemodynamics), iii) tolerating solid diet (no nausea, no vomiting), iv) return of bowel function (passage of flatus/stools), v) full mobilization.
8. Telephone number for consultation available 24/7. Follow up outpatient appointment in 1–2 weeks.

**Table 5:** Donors’ Outcomes: Surgical Mortality.

Authors, Year	Surgical Mortality	
	ERAS	Standard Care
**Non-Randomized Studies**		
Ricotta, C., et al., 2019 [12]	0/21(0%)	0/55 (0%)
Rege A et al., 2016 [19]	0/39 (0%)	0/40 (0%)
Waits S, et al., 2015 [20]	0/60 (0%)	0/60 (0%)
Kuo P., et al., 2000 [17]	0/41 (0%)	0/27 (0%)
**Randomized Clinical Trials**		
Campsen, J., et al., 2019 [11]	0/33 (0%)	0/29 (0%)
Ahmed M Mansour, et al., 2017 [13]	0/110 (0%)	0/109 (0%)
Campsen, J., et al., 2017 [14]	0/5 (0%)	0/5 (0%)
Alberts V. P, et al., 2014 [15]	0/26 (0%)	0/26 (0%)

**Table 6:** Donors’ Outcomes: Surgical Morbidity.

Authors, Year	Post-op Complications									
	Overall		Clavien Dindo I–II		Clavien Dindo III–V		Δhematocrit (Pre-op–Post-op)		Post-op Blood Transfusion	
	ERAS	Standard Care	ERAS	Standard Care	ERAS	Standard Care	ERAS	Standard Care	ERAS	Standard Care
**Non-Randomized Studies**										
Brown, T et al., 2020 [10]	Not reported	Not reported	Not reported	Not reported	Not reported	Not reported	10.1% ± 6.1% *	10.8% ± 5.6% *	0/24 (0%)	0/24 (0%)
Kuo P., et al., 2000 [17]	0/41 (0%)	4/27 (14.8%)	0/41 (0%)	4/27 (4.8%)	0/41 (0%)	0/27 (0%)	Not reported	Not reported	Not reported	Not reported
**Randomized Clinical Trials**										
Campsen, J., et al., 2019 [11]	Not reported	Not reported	Not reported	Not reported	Not reported	Not reported	6.3 ± 2.1	4.3 ± 2.5	0/33 (0%)	0/29 (0%)
Ahmed M Mansour, et al., 2017 [13]	15/110 (13.6%)	39/109 (35.7%)	15/110 (13.6%)	39/109 (35.7%)	0/110 (0%)	0/109 (0%)	Not reported	Not reported	2/110 (1.8%)	1/110 (0.9%)
Campsen, J., et al., 2017 [14]	Not reported	Not reported	Not reported	Not reported	Not reported	Not reported	6.2 ± 2.05	6.3 ± 4	0/5 (0%)	0/5 (0%)
Alberts V. P, et al., 2014 [15]	1/26 (4%)	1/26 (4%)	1/26 (4%)	1/26 (4%)	0/26 (0%)	0/26 (0%)	Not reported	Not reported	Not reported	Not reported

* percentage of haematocrit drop reported.

**Table 7:** Donors’ Outcomes: Length of Stay and Readmissions.

Authors, Year	LOS (in Days)		Readmissions		Reasons for Readmissions	
	ERAS	Standard Care	ERAS	Standard Care	ERAS	Standard Care
**Non-Randomized Studies**						
Brown, T et al., 2020 [10]	2.58 ± 1.02	2.96 ± 1.20	0/44 (0%)	3/22 (13.6%)	Not Reported	Not Reported
Ricotta, C., et al., 2019 [12]	5 (4–10)	5 (3–15)	0/21 (0%)	0/76 (0%)	Not Reported	Not Reported
Rege A et al., 2016 [19]	1 (1–1)	2 (1–7)	5/39 (12.8%)	11/40 (27.5%)	GI dysfunction	Not Reported
Waits S, et al., 2015 [20]	1 (1–5)	2 (1–5)	3/60 (5%)	4/60 (6.6%)	prolonged ileus, wound infection	Not Reported
Panaro F., et al., 2011 [18]	3.2	4.5	Not Reported	Not Reported	Not Reported	Not Reported
Milan Z., et al., 2011 [16]	3.7	4.7	Not Reported	Not Reported	Not Reported	Not Reported
Kuo P., et al., 2000 [17]	1 ± 0.1	2.6 ± 0.2	1/41 (2.4%)	0/27 (0%)	Nausea	Not Reported
**Randomized Clinical Trials**						
Campsen, J., et al., 2019 [11]	2.14 ±0.2	2.39 ±0.3	0/33 (0%)	0/29 (0%)	Not Reported	Not Reported
Ahmed M Mansour, et al., 2017 [13]	2.8 ± 1.0	3.9 ±1.7	Not Reported	Not Reported	Not Reported	Not Reported
Campsen, J., et al., 2017 [14]	2.58 ± 0.51	2.65 ± 0.56	Not Reported	Not Reported	Not Reported	Not Reported
Alberts V. P, et al., 2014 [15]	3 ±1	4 ± 1	Not Reported	Not Reported	Not Reported	Not Reported

**Table 8:** Donors’ Outcomes: QoL-Postoperative Pain.

Authors, Year	Pain Scores 0–24 h Post-op		Pain Scores 24–48 h Post -op		Morphine Requirements 0–24 h Post-op (in mg)		Morphine Requirements 24–48 h Post-op (in mg)		Cumulative Morphine Requirements 0–48 h Post-op or throughout Hospital Stay (in mg)	
	ERAS	Standard Care	ERAS	Standard Care	ERAS	Standard Care	ERAS	Standard Care	ERAS	Standard Care
**Non-Randomized Studies**										
Brown, T et al., 2020 [10]	2.7/10	2.9/10	1.8/10	1.9/10	11.59 ± 10.01	21.1 ± 8.55	14.83 ± 13.76	23.85 ± 10.85	26.42 ± 17.07	44.95 ± 13.81
Rege A et al., 2016 [19]	3/10 (2/10–6/10)	7/10 (4/40–8/10)	Not Reported	Not Reported	Not Reported	Not Reported	Not Reported	Not Reported	Not Reported	Not Reported
Waits S, et al., 2015 [20]	3.87/10	3.97/10	Not Reported	Not Reported	Not Reported	Not Reported	Not Reported	Not Reported	21.6	45.6
Panaro F., et al., 2011 [18]	0/10	1.6/10	0/10	1.3/10	6.4	12.4	4.9	13.6	11.3	26
Milan Z., et al., 2011 [16]	1.0/3	1.0/3	0/3	1.0/3	Not Reported	Not Reported	Not Reported	Not Reported	Not Reported	Not Reported
**Randomized Clinical Trials**										
Campsen, J., et al., 2019 [11]	Not Reported	Not Reported	Not Reported	Not Reported	Not Reported	Not Reported	Not Reported	Not Reported	27 ±14.5	45 ±22.5
Ahmed M Mansour, et al., 2017 [13]	1.81/10 ± 0.62/10	2.22/10 ± 0.56/10	4.04/10 ± 1.86/10	7.27/10 ±1.01/10	Not Reported	Not Reported	Not Reported	Not Reported	Not Reported	Not Reported
Campsen, J., et al., 2017 [14]	Not Reported	Not Reported	Not Reported	Not Reported	Not Reported	Not Reported	Not Reported	Not Reported	143.6 ± 104.2	192.9 ±118
Alberts V. P, et al., 2014 [15]	1.2/10 ± 1/10	2.8/10 ± 2.0/10	3.2/10 ± 2.2/10	2.0/10 ± 1.9/10	Not Reported	Not Reported	Not Reported	Not Reported	Not Reported	Not Reported

**Table 9:** Donors’ Outcomes: QoL-SF-36 Questionaire.

SF-36			Ahmed M Mansour, et al., 2017 [13]	Alberts V. P, et al., 2014 [15]
Physical Function	ERAS	Pre op	96.8 ± 9.1	97 ± 1.5
		Post op	89.3 ± 17.9	75 ±3.0
	Standard Care	Pre op	95.2 ± 12.7	97 ± 2
		Post op	79.7 ± 16.1	73 ±2.6
Role physical	ERAS	Pre op	91.6 ± 10.7	96 ± 4.5
		Post op	75.0 ± 31.7	39± 9
	Standard Care	Pre op	87.9 ± 15.0	93 ± 5
		Post op	78.3 ± 15.7	27.5 ± 5.5
Bodily pain	ERAS	Pre op	89.4 ± 14.8	94 ± 4.5
		Post op	85.8 ± 15.9	75 ± 5
	Standard Care	Pre op	90.2 +16.3	90.5 ± 3.5
		Post op	71.0 ± 16.2	65.5 ±5.5
General Health	ERAS	Pre op	88.0 ± 17.4	84 ± 4
		Post op	81.6 ± 16.8	79 ± 5
	Standard Care	Pre op	89.4 ± 15.7	85 ± 4.5
		Post op	74.1 ± 18.1	84.8 ±4.9
Vitality	ERAS	Pre op	78.4 ± 16.7	90 ± 2
		Post op	62.3 ± 18.5	66 ± 4
	Standard Care	Pre op	77.4 ± 12.5	83.2 ± 6.3
		Post op	63.9 ± 18.0	58 ± 3
Social Functioning	ERAS	Pre op	94.8 ± 15.5	97 ± 2.3
		Post op	81.3 ± 21.4	80 ± 4
	Standard Care	Pre op	91.5 ± 16.9	97 ± 2
		Post op	84.6 ± 17.5	75 ± 5
Role emotional	ERAS	Pre op	89.7 +14.4	94 ± 5
		Post op	82.5 ± 24.7	90 ± 8
	Standard Care	Pre op	91.6 ± 11.3	99 ± 1
		Post op	72.6 ± 11.3	80 ± 8.5
Mental Health	ERAS	Pre op	85.5 ± 16.0	86 ± 2
		Post op	81.8 ± 11.0	89 ±1
	Standard Care	Pre op	88.5 ± 19.0	89 ± 2
		Post op	85.8 ± 16.6	89 ± 2

## Supplementary Materials

The following are available online at https://www.mdpi.com/2077-0383/10/1/21/s1. Table S1: Medline Database Search. Table S2: Embase Database Search. File S1: ROBINS-I tool: Brown, T., et al. 2020. “Introduction of an enhanced recovery protocol into a laparoscopic living donor nephrectomy programme.” File S2: Rob 2 tool: Campsen, J., et al. 2019. “Prospective, double-blind, randomized clinical trial comparing an ERAS pathway with ketorolac and pregabalin versus standard of care plus placebo during live donor nephrectomy for kidney transplant.” File S3: ROBINS-I tool: Ricotta, C., et al., 2019. “Enhanced Recovery after Implementation of Surgery Protocol in Living Kidney Donors: The ISMETT Experience.” File S4: Rob 2 tool: Ahmed M. Mansour, et al., 2017. “Enhanced Recovery Open vs. Laparoscopic Left Donor Nephrectomy: A Randomized Controlled Trial” File S5: Rob 2 tool: Campsen, J., et al., 2017. “TORPEDO: Prospective, double blind, randomized clinical trial comparing the use of Ketorolac verse placebo during live donor nephrectomy for kidney transplant.” File S6: Rob 2 tool: Alberts, V., 2014. “Fast track hand-assisted laparoscopic donor nephrectomy: a randomized clinical trial” File S7: ROBINS-I tool: Millan, Z., et al., 2011. “Is Single-Shot Epidural Analgesia More Effective Than Morphine Patient-Controlled Analgesia for Donor Nephrectomy?” File S8: ROBINS-I tool: Paul C. Kuo et al., 2000. “Laparoscopic Donor Nephrectomy With a 23-Hour Stay: A New Standard for Transplantation Surgery.” File S9: ROBINS-I tool: Panaro, F., et al., 2011. “Continuous Infusion of Local Anesthesia After Living Donor Nephrectomy: A Comparative Analysis” File S10: ROBINS-I tool: Rege A., et al., 2016. Could the Use of an Enhanced Recovery Protocol in Laparoscopic Donor Nephrectomy be an Incentive for Live Kidney Donation? File S11: ROBINS-I tool: Waits S., et al., 2015. “Building the Case for Enhanced Recovery Protocols in Living Kidney Donors.” S12: Subgroup meta-analyses: Nonrandomized and Randomized Data.
