# Supplementary material for: Feasibility, Safety and Efficacy of Enhanced Recovery after Living Donor Nephrectomy: Systematic Review and Meta-Analysis of Randomized Controlled Trials
Source: J Clin Med. 2020 Dec 23;10(1):21. doi: 10.3390/jcm10010021 (PMC7795400; doi:10.3390/jcm10010021)
Supplement: Supplementary file 1 [file jcm-10-00021-s001.zip › Supplementary Files/Supplementary File S8.docx]

The Risk Of Bias In Non-randomized Studies – of Interventions (ROBINS-I) assessment tool

(version for cohort-type studies)

**Version 19 September 2016**


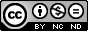


This work is licensed under a [Creative Commons Attribution-NonCommercial-NoDerivatives 4.0 International License](http://creativecommons.org/licenses/by-nc-nd/4.0/).

# ROBINS-I tool (Stage I): At protocol stage

## Specify the review question

| Participants | Living kidney donors; Renal transplant Recipients |
| --- | --- |
| Experimental intervention | At least 2 or more sequential Enhanced Recovery After Surgery Interventions |
| Comparator | Standard Care |
| Outcomes | 1.post-op complications in donors and in recipients  2. post-op mortality in donors and in recipients  3. Length of Stay in donors and in recipients  4.Readmission rates up to 90 days post discharge in donors and in recipients  5. Reasons for readmissions in donors and in recipients.  6. Adverse events in donors or recipients  7.Quality of Life Data (Donors) (available qualitative/quantitative data on six domains: patients’ support, comfort, emotions, physical independence, pain and satisfaction during the recovery period – at any point up to 90 days post hospital discharge) |

## List the confounding domains relevant to all or most studies

| 1. Donors’ demographics (mainly age but race, gender and socioeconomic status are relevant as well)  2. Donors’ comorbidities (mainly Asthma/ COPD, HTN, dyslipidemia, glucose intolerance previous abdominal surgeries / laparotomies but any comorbidity is relevant in living donors. In deceased donors: any comorbidity)  3.Donors’ BMI  3.Donors’ smoking history prior to enrollment  4.Donors’ kidney function prior to enrollment  6. Number of Marginal/extended criteria donors  7.Recipients demographics (Mainly Age but race, gender and socioeconomic status are relevant as well)  8.Recipients Comorbidities (Mainly Asthma/COPD, diabetes, PVD, CCF, cebrovascular disease, liver disease, recurrent UTIs and previous abdominal surgeries/ laparotomies but any comorbidity is relevant)  9.Recipients’ BMI  10.Recipients smoking history prior to enrollment  11.Renal replacement therapy  12.Time in dialysis  13.Previous kidney transplant  14.Number of living and number of deceased donors  15.ABO incompatibility  16.Positive tissue crossmatch  17.HLA mismatch  18.Warm ischemia time  19.Cold ischemia time  20.Overall ischemia time  21.Left or right donor nephrectomy/ kidney transplant |
| --- |

##

## List co-interventions that could be different between intervention groups and that could impact on outcomes

| Any intervention in the graft  Immunosuppression regime  Any intervention outside the recovery pathway |
| --- |

# ROBINS-I tool (Stage II):Kuo, P. et al, 2000.

## Specify a target randomized trial specific to the study

| Design | Cluster randomized |
| --- | --- |
| Participants | Adult Living Kidney Donors |
| Experimental intervention | **Enhanced Recovery After Surgery**  Preoperative:i. pre-op patient education ii. informed consent  Intraoperative: i. laparoscopic living donor nephrectomy ii. mannitol, furosemide, ondastetron, ketorolac iii. heparin before clamp and protamin after clamp release iv. liberal fluids administration with target diuresis 1ml/kg.h v. urinary catheter vi. Antiemetics at the end of the operation  Post-operative: i. IV abx for 24 h ii. oxycodone PRN and regular paracetamol iii. removal of urinary catheter on POD0 iv. free fluids on POD0 v. mobilization on POD0 vi.solid food on POD1 |
| Comparator | **Standard Care**  Preoperative:i. pre-op patient education ii. informed consent  Intraoperative: i. laparoscopic living donor nephrectomy ii. mannitol and furosemide iii. heparin before clamp and protamin after clamp release iv. liberal fluids administration with target diuresis 1ml/kg.h v. urinary catheter vi. Antiemetics at the end of the operation  Post-operative: i. IV abx for 24 h ii. morphine PCA iii. removal of urinary catheter on POD0 iv. free fluids on POD0 v. solid food on POD1 |

## Is your aim for this study…?

| x | to assess the effect of *assignment to* intervention |
| --- | --- |
| □ | to assess the effect of *starting and adhering to* intervention |

## Specify the outcome

Specify which outcome is being assessed for risk of bias (typically from among those earmarked for the Summary of Findings table). Specify whether this is a proposed benefit or harm of intervention.

| Donors’ Length of stay ; Donors’ overall complications (Clavien Dindo I and II and >=III) ; Donors’ surgical mortality ; Donors’ Readmission Rates ; Donors’ Reasons for Readmissions ; Dnors’ Satisfaction ; Recipients surgical mortality ; Recipients’ overall complications (Clavien Dindo I and II and >=III) ; Recipients’ Dealyed Graft Function ; Recipients’ graft survival up to 1 year |
| --- |

## Preliminary consideration of confounders

Complete a row for each important confounding domain (i) listed in the review protocol; and (ii) relevant to the setting of this particular study, or which the study authors identified as potentially important.

#### “Important” confounding domains are those for which, in the context of this study, adjustment is expected to lead to a clinically important change in the estimated effect of the intervention. “Validity” refers to whether the confounding variable or variables fully measure the domain, while “reliability” refers to the precision of the measurement (more measurement error means less reliability).

| **(i) Confounding domains listed in the review protocol** | | | | |
| --- | --- | --- | --- | --- |
| Confounding domain | Measured variable(s) | Is there evidence that controlling for this variable was unnecessary?* | Is the confounding domain measured validly and reliably by this variable (or these variables)? | OPTIONAL: Is failure to adjust for this variable (alone) expected to favour the experimental intervention or the comparator? |
| Donors’ demographics | Gender | No | No | No information |
| Donors’ comorbidities | Not assessed | No | No | No information |
| Donors’ BMI | Not assessed | No | No | No information |
| Donors’ smoking history prior to enrollment | Not assessed | No | No | No information |
| Donors’ kidney function prior to enrollment | Not assessed | No | No | No information |
| Number of Marginal/extended criteria donors | Not assessed | No | No | No information |
| Recipients demographics | Not assessed | No | No | No information |
| Recipients Comorbidities | Not assessed | No | No | No information |
| Recipients’ BMI | Not assessed | No | No | No information |
| Confounding domain | Measured variable(s) | Is there evidence that controlling for this variable was unnecessary?* | Is the confounding domain measured validly and reliably by this variable (or these variables)? | OPTIONAL: Is failure to adjust for this variable (alone) expected to favour the experimental intervention or the comparator? |
| Recipients smoking history prior to enrollment | Not assessed | No | No | No information |
| Renal replacement therapy | Not assessed | No | No | No information |
| Time in dialysis | Not assessed | No | No | No information |
| Previous kidney transplant | Not assessed | No | No | No information |
| Number of living and number of deceased donors | Only Living Donors | Yes | Yes | - |
| ABO incompatibility | Not assessed | No | No | No information |
| Positive tissue crossmatch | Not assessed | No | No | No information |
| HLA mismatch | Not assessed | No | No | No information |
| Warm ischemia time | Not assessed | No | No | No information |
| Cold ischemia time | Not assessed | No | No | No information |
| Overall ischemia time | Not assessed | No | No | No information |
| Left or right donor nephrectomy/ kidney transplant | Not assessed | No | No | No information |

| **(ii) Additional confounding domains relevant to the setting of this particular study, or which the study authors identified as important** | | | | |
| --- | --- | --- | --- | --- |
| Confounding domain | Measured variable(s) | Is there evidence that controlling for this variable was unnecessary?* | Is the confounding domain measured validly and reliably by this variable (or these variables)? | OPTIONAL: Is failure to adjust for this variable (alone) expected to favour the experimental intervention or the comparator? |
|  |  |  | Yes / No / No information | Favour experimental / Favour comparator / No information |
|  |  |  |  |  |
|  |  |  |  |  |
|  |  |  |  |  |
|  |  |  |  |  |
|  |  |  |  |  |
|  |  |  |  |  |
|  |  |  |  |  |
|  |  |  |  |  |
|  |  |  |  |  |

* In the context of a particular study, variables can be demonstrated not to be confounders and so not included in the analysis: (a) if they are not predictive of the outcome; (b) if they are not predictive of intervention; or (c) because adjustment makes no or minimal difference to the estimated effect of the primary parameter. Note that “no statistically significant association” is not the same as “not predictive”.

## Preliminary consideration of co-interventions

Complete a row for each important co-intervention (i) listed in the review protocol; and (ii) relevant to the setting of this particular study, or which the study authors identified as important.

#### “Important” co-interventions are those for which, in the context of this study, adjustment is expected to lead to a clinically important change in the estimated effect of the intervention.

| **(i) Co-interventions listed in the review protocol** | | |
| --- | --- | --- |
| Co-intervention | Is there evidence that controlling for this co-intervention was unnecessary (e.g. because it was not administered)? | Is presence of this co-intervention likely to favour outcomes in the experimental intervention or the comparator |
| Any intervention in the graft | Yes |  |
| Immunosuppression Regime | Yes |  |
| Any intervention outside the recovery pathway | Yes |  |
|  |  |  |

| **(ii) Additional co-interventions relevant to the setting of this particular study, or which the study authors identified as important** | | |
| --- | --- | --- |
| Co-intervention | Is there evidence that controlling for this co-intervention was unnecessary (e.g. because it was not administered)? | Is presence of this co-intervention likely to favour outcomes in the experimental intervention or the comparator |
|  |  | Favour experimental / Favour comparator / No information |
|  |  | Favour experimental / Favour comparator / No information |
|  |  | Favour experimental / Favour comparator / No information |
|  |  | Favour experimental / Favour comparator / No information |

## Risk of bias assessment

Responses underlined in green are potential markers for low risk of bias, and responses in red are potential markers for a risk of bias. Where questions relate only to sign posts to other questions, no formatting is used.

|  | **Signalling questions** | **Description** | **Response options** |
| --- | --- | --- | --- |
| **Bias due to confounding** | | | |
|  | 1.1 Is there potential for confounding of the effect of intervention in this study?  **If N/PN to 1.1:** the study can be considered to be at low risk of bias due to confounding and no further signalling questions need be considered |  | PY |
|  | **If Y/PY to 1.1**: determine whether there is a need to assess time-varying confounding: |  |  |
|  | 1.2. Was the analysis based on splitting participants’ follow up time according to intervention received?  **If N/PN**, answer questions relating to baseline confounding (1.4 to 1.6)  **If Y/PY**, go to question 1.3. |  | Y |
|  | 1.3. Were intervention discontinuations or switches likely to be related to factors that are prognostic for the outcome?  **If N/PN**, answer questions relating to baseline confounding (1.4 to 1.6)  **If Y/PY**, answer questions relating to both baseline and time-varying confounding (1.7 and 1.8) |  | PN |

|  | **Questions relating to baseline confounding only** | | |
| --- | --- | --- | --- |
|  | 1.4. Did the authors use an appropriate analysis method that controlled for all the important confounding domains? |  | N |
|  | 1.5. **If Y/PY to 1.4**: Were confounding domains that were controlled for measured validly and reliably by the variables available in this study? |  | PN |
|  | 1.6. Did the authors control for any post-intervention variables that could have been affected by the intervention? |  | NA |
|  | **Questions relating to baseline and time-varying confounding** | |  |
|  | 1.7. Did the authors use an appropriate analysis method that controlled for all the important confounding domains and for time-varying confounding? |  | NA |
|  | 1.8. **If Y/PY to 1.7**: Were confounding domains that were controlled for measured validly and reliably by the variables available in this study? |  | NA |
|  | **Risk of bias judgement** |  | Critical |
|  | Optional: What is the predicted direction of bias due to confounding? |  | Unpredictable |

| **Bias in selection of participants into the study** | | | |
| --- | --- | --- | --- |
|  | 2.1. Was selection of participants into the study (or into the analysis) based on participant characteristics observed after the start of intervention?  **If N/PN to 2.1:** go to 2.4 |  | PN |
|  | 2.2. **If Y/PY to 2.1**: Were the post-intervention variables that influenced selection likely to be associated with intervention?  2.3 **If Y/PY to 2.2**: Were the post-intervention variables that influenced selection likely to be influenced by the outcome or a cause of the outcome? |  | NA  NA |
|  | 2.4. Do start of follow-up and start of intervention coincide for most participants? |  | PN |
|  | 2.5. **If Y/PY to 2.2 and 2.3, or N/PN to 2.4**: Were adjustment techniques used that are likely to correct for the presence of selection biases? |  | PN |
|  | **Risk of bias judgement** |  | Serious |
|  | Optional: What is the predicted direction of bias due to selection of participants into the study? |  | Unpredictable |

| **Bias in classification of interventions** | | | |
| --- | --- | --- | --- |
|  | 3.1 Were intervention groups clearly defined? |  | Y |
|  | 3.2 Was the information used to define intervention groups recorded at the start of the intervention? |  | PY |
|  | 3.3 Could classification of intervention status have been affected by knowledge of the outcome or risk of the outcome? |  | PN |
|  | **Risk of bias judgement** |  | Low |
|  | Optional: What is the predicted direction of bias due to classification of interventions? |  | Towards null |

| **Bias due to deviations from intended interventions** | | | |
| --- | --- | --- | --- |
|  | **If your aim for this study is to assess the effect of assignment to intervention, answer questions 4.1 and 4.2** | |  |
|  | 4.1. Were there deviations from the intended intervention beyond what would be expected in usual practice? |  | PN |
|  | 4.2. **If Y/PY to 4.1**: Were these deviations from intended intervention unbalanced between groups *and* likely to have affected the outcome? |  | NA |
|  | **If your aim for this study is to assess the effect of starting and adhering to intervention, answer questions 4.3 to 4.6** | |  |
|  | 4.3. Were important co-interventions balanced across intervention groups? |  |  |
|  | 4.4. Was the intervention implemented successfully for most participants? |  |  |
|  | 4.5. Did study participants adhere to the assigned intervention regimen? |  |  |
|  | 4.6. **If N/PN to 4.3, 4.4 or 4.5**: Was an appropriate analysis used to estimate the effect of starting and adhering to the intervention? |  |  |
|  | **Risk of bias judgement** |  | Low |
|  | Optional: What is the predicted direction of bias due to deviations from the intended interventions? |  | Towards null |

| **Bias due to missing data** | | | |
| --- | --- | --- | --- |
|  | 5.1 Were outcome data available for all, or nearly all, participants? |  | Y |
|  | 5.2 Were participants excluded due to missing data on intervention status? |  | PN |
|  | 5.3 Were participants excluded due to missing data on other variables needed for the analysis? |  | PN |
|  | 5.4 **If PN/N to 5.1, or Y/PY to 5.2 or 5.3**: Are the proportion of participants and reasons for missing data similar across interventions? |  | NA |
|  | 5.5 **If PN/N to 5.1, or Y/PY to 5.2 or 5.3**: Is there evidence that results were robust to the presence of missing data? |  | NA |
|  | **Risk of bias judgement** |  | Low |
|  | Optional: What is the predicted direction of bias due to missing data? |  | Towards null |

| **Bias in measurement of outcomes** | | | |
| --- | --- | --- | --- |
|  | 6.1 Could the outcome measure have been influenced by knowledge of the intervention received? |  | PN |
|  | 6.2 Were outcome assessors aware of the intervention received by study participants? |  | Y |
|  | 6.3 Were the methods of outcome assessment comparable across intervention groups? |  | Y |
|  | 6.4 Were any systematic errors in measurement of the outcome related to intervention received? |  | N |
|  | **Risk of bias judgement** |  | Low |
|  | Optional: What is the predicted direction of bias due to measurement of outcomes? |  | Towards null |

| **Bias in selection of the reported result** | | | |
| --- | --- | --- | --- |
|  | Is the reported effect estimate likely to be selected, on the basis of the results, from... |  |  |
|  | 7.1. ... multiple outcome *measurements* within the outcome domain? |  | PN |
|  | 7.2 ... multiple *analyses* of the intervention-outcome relationship? |  | PN |
|  | 7.3 ... different *subgroups*? |  | PN |
|  | **Risk of bias judgement** |  | Low |
|  | Optional: What is the predicted direction of bias due to selection of the reported result? |  | Towards null |

| **Overall bias** | | | |
| --- | --- | --- | --- |
|  | **Risk of bias judgement** |  | Critical |
|  | Optional: What is the overall predicted direction of bias for this outcome? |  | Unpredictable |


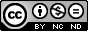


This work is licensed under a [Creative Commons Attribution-NonCommercial-NoDerivatives 4.0 International License](http://creativecommons.org/licenses/by-nc-nd/4.0/).
