# Supplementary material for: Feasibility, Safety and Efficacy of Enhanced Recovery after Living Donor Nephrectomy: Systematic Review and Meta-Analysis of Randomized Controlled Trials
Source: J Clin Med. 2020 Dec 23;10(1):21. doi: 10.3390/jcm10010021 (PMC7795400; doi:10.3390/jcm10010021)
Supplement: Supplementary file 1 [file jcm-10-00021-s001.zip › Supplementary Files/Supplementary Tables - conversion.pdf]

Table S1. Medline Database Search.

1. Kidney Transplantation/ae, ed, is, mt, mo, nu, ph, px, rh, st, sn [Adverse Effects, Education, Instrumentation, Methods, Mortality, Nursing, Physiology, Psychology, Rehabilitation, Standards, Statistics & Numerical Data]
2. Nephrectomy/ae, co, ed, is, mt, mo, nu, ph, px, rh, st, sn [Adverse Effects, Complications, Education, Instrumentation, Methods, Mortality, Nursing, Physiology, Psychology, Rehabilitation, Standards, Statistics & Numerical Data]
3. laparoscopic living donor nephrectomy.mp.
4. living kidney donors.mp.
5. renal transplant recipients.mp.
6. kidney transplant recipients.mp.
7. 1 or 2 or 3 or 4 or 5 or 6
8. "Recovery of Function"/ or enhanced recovery after surgery.mp. or Enhanced Recovery After Surgery/
9. enhanced recovery protocols.mp.
10. enhanced recovery.mp.
11. ERAS.mp.
12. Time Factors/
13. Fast-Track.mp.
14. Adult/
15. Living Donors/
16. enhanced recovery programs.mp.
17. non enhanced recovery.mp.
18. non ERAS.mp.
19. standard recovery.mp.
20. standard treatment.mp.
21. standard care.mp.
22. "Standard of Care"/og, st, sn [Organization & Administration, Standards, Statistics & Numerical Data]
23. control groups/
24. 14 or 15 or 16 or 17 or 18 or 19 or 20 or 21 or 22 or 23
25. "Length of Stay"/sn [Statistics & Numerical Data]
26. Patient Readmission/sn [Statistics & Numerical Data]
27. Mortality/
28. Morbidity/sn, td [Statistics & Numerical Data, Trends]
29. Postoperative Complications/
30. treatment outcome/
31. donor readmission rates.mp.
32. enhanced recovery after surgery.mp. or Enhanced Recovery After Surgery/
33. Pain, Postoperative/co, dt, ep, et, mo, nu, pp, pc, px, rh, su, th [Complications, Drug Therapy, Epidemiology, Etiology, Mortality, Nursing, Physiopathology, Prevention & Control, Psychology, Rehabilitation, Surgery, Therapy]
34. "Quality of Life"/
35. 25 or 26 or 27 or 28 or 29 or 30 or 31 or 32 or 33 or 34
36. 8 or 9 or 10 or 11 or 12 or 13
37. 7 and 24 and 35 and 36

Table S2. Embase Database Search.

1. kidney transplantation/ae, co, ep, rh, su, th [Adverse Drug Reaction, Complication, Epidemiology, Rehabilitation, Surgery, Therapy]
2. nephrectomy/ae, co, et, rh [Adverse Drug Reaction, Complication, Etiology, Rehabilitation]
3. kidney donor/ or living kidney donors.mp.
4. kidney transplant recipients.mp.
5. renal transplant recipients.mp.
6. kidney graft/
7. kidney graft recipients.mp.
8. 1 or 2 or 3 or 4 or 5 or 6 or 7
9. enhanced recovery after surgery.mp. or enhanced recovery after surgery/
10. ERAS.mp.
11. enhanced recovery.mp.
12. enhanced recovery protocols.mp.
13. enhanced recovery programs.mp.
14. Fast Track.mp.
15. 9 or 10 or 11 or 12 or 13 or 14
16. adult/
17. living donor/
18. donor/
19. kidney recipient.mp.
20. non enhanced recovery.mp.
21. non ERAS.mp.
22. standard treatment.mp.
23. standard recovery.mp.
24. standard of care.mp.
25. 16 or 17 or 18 or 19 or 20 or 21 or 22 or 23 or 24
26. morbidity/
27. postoperative complication/co, dm, dt, ep, et, pc, rh, si, su, th [Complication, Disease Management, Drug Therapy, Epidemiology, Etiology, Prevention, Rehabilitation, Side Effect, Surgery, Therapy]
28. mortality rate/ or hospital mortality/ or all cause mortality/ or mortality/ or surgical mortality/
29. "length of stay"/
30. hospital readmission/
31. "quality of life"/
32. treatment outcome/
33. 26 or 27 or 28 or 29 or 30 or 31 or 32
34. 8 and 15 and 25 and 33
